# Supplementary material for: Contrasting effects of increasing dissolved iron on photosynthesis and O2 availability in the gastric cavity of two Mediterranean corals
Source: PeerJ. 2024 Apr 29;12:e17259. doi: 10.7717/peerj.17259 (PMC11064864; doi:10.7717/peerj.17259)
Supplement: Supplemental Information 1 [file peerj-12-17259-s001.docx]

**Increasing dissolved iron limits photosynthesis and reduces the O_2_ availability in the gastric cavity of two Mediterranean corals.**

**Supplementary Materials**


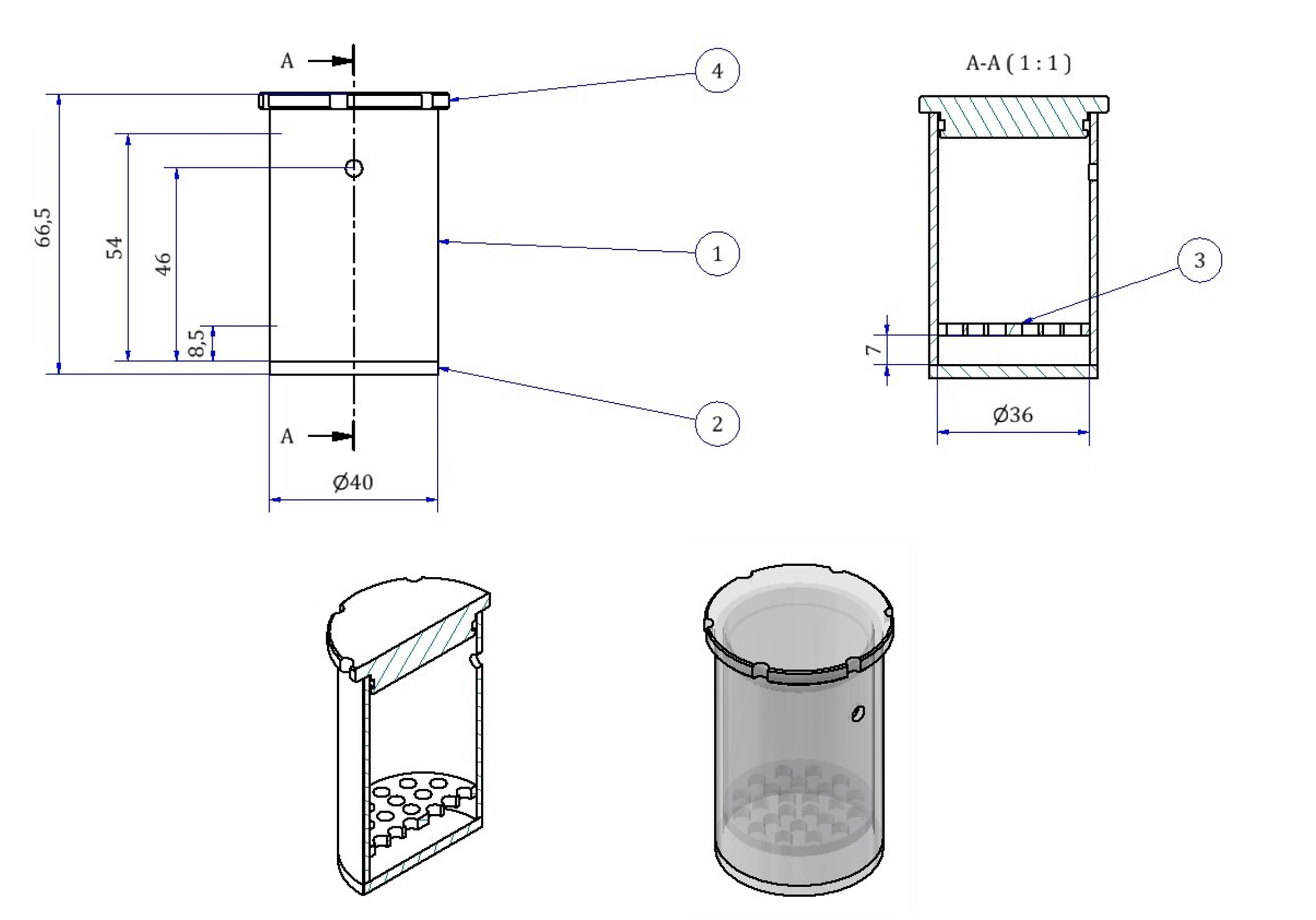


**Figure S1.** Schematic representation of the custom-made respirometry chambers. 1) chamber, 2) bottom surface; 3) shelf for magnetic stirrer; 4) lid.

**Table S1.** Resume of Kruskal-Wallis test for respiration, net photosynthesis and gross photosynthesis; analysis of variance (ANOVA) for P:R and maximum PSII quantum yield (Fv/Fm).

| **Kruskal-Wallis test** | |  |  |  |  |  |  |
| --- | --- | --- | --- | --- | --- | --- | --- |
| **Species** | **Parameter** | **N** | **Chi-square** | **Df** | **p-value** |  |  |
| *C. caespitosa* | Respiration | 24 | 11.6 | 3 | < 0.01 |  |  |
|  | Net Photosynthesis | 24 | 12.2 | 3 | < 0.01 |  |  |
|  | Gross Photosynthesis | 24 | 11.8 | 3 | < 0.01 |  |  |
| *O. patagonica* | Respiration | 24 | 19.1 | 3 | < 0.01 |  |  |
|  | Net Photosynthesis | 24 | 8.54 | 3 | < 0.05 |  |  |
|  | Gross Photosynthesis | 24 | 19.4 | 3 | < 0.01 |  |  |
| **ANOVA test** |  |  |  |  |  |  |  |
| **Species** | **Parameters** |  | **Df** | **S.S.** | **Mean Sq.** | **F-value** | **p-value** |
| *C. caespitosa* | P:R | Condition | 3 | 0.01 | 0.003 | 0.93 | *n.s.* |
|  |  | Residuals | 20 | 0.074 | 0.004 |  |  |
|  | Fv/Fm | Condition | 3 | 0.012 | 0.004 | 3.83 | < 0.05 |
|  |  | Residuals | 13 | 0.014 | 0.001 |  |  |
| *O. patagonica* | P:R | Condition | 3 | 0.187 | 0.062 | 8.29 | < 0.01 |
|  |  | Residuals | 20 | 0.151 | 0.008 |  |  |
|  | Fv/Fm | Condition | 3 | 0.352 | 0.117 | 10.94 | < 0.01 |
|  |  | Residuals | 13 | 0.14 | 0.011 |  |  |

**Table S2**. Summary of the Kruskal-Wallis test for the measurements of variable chlorophyll fluorescence under different conditions. Chi-squared = test statistic H; Df = degrees of freedom.

| **Species** | **Parameter** | **Chi-squared** | **Df** | **p-value** |
| --- | --- | --- | --- | --- |
| *C. caespitosa* | F0 | 92.187 | 3 | < 0.01 |
|  | Fm | 20.085 | 3 | < 0.01 |
|  | ETR(II) | 9.225 | 3 | < 0.05 |
|  | Y(II) | 7.054 | 3 | *n.s.* |
| *O. patagonica* | F0 | 110.06 | 3 | < 0.01 |
|  | Fm | 17.625 | 3 | < 0.01 |
|  | ETR(II) | 4.796 | 3 | *n.s.* |
|  | Y(II) | 15.109 | 3 | < 0.01 |

**Table S3**. Summary of the Kruskal-Wallis test for the measurements of oxygen availability within the gastric cavity of *C. caespitosa* and *O. patagonica* under different conditions (dark, light, and overnight). Chi-squared = test statistic H; Df = degrees of freedom.

| **Species** | **Condition** | **Chi-squared** | **Df** | **p-value** |
| --- | --- | --- | --- | --- |
| *C. caespitosa* | Dark | 115 | 3 | < 0.01 |
|  | Light | 32.9 | 3 | < 0.01 |
|  | Overnight | 1475 | 3 | < 0.01 |
| *O. patagonica* | Dark | 163 | 3 | < 0.01 |
|  | Light | 11.1 | 3 | < 0.05 |
|  | Overnight | 2140 | 3 | < 0.01 |

**Table S4.** Summary of the linear regression model for the estimation of the O_2_ level using contraction time, condition, and species are predictors. Residual standard error: 44.22 on 6257 D.f.; Multiple R-squared: 0.4006; Adjusted R-squared: 0.4002; F-statistic: 1046 on 4 and 6257 D.f.; p-value: < 2.2e-16.

| *Residuals* |  |  |  |  |
| --- | --- | --- | --- | --- |
| **Min** | **1Q** | **Median** | **3Q** | **Max** |
| -129.037 | -38.811 | -4.528 | 31.253 | 148.26 |
| *Coefficients* | **Estimate** | **Std. Error** | **T value** | **p-value** |
| Intercept | 58.819 | 1.505 | 39.09 | < 0.01 |
| Contraction Time | -0.586 | 0.057 | -10.30 | < 0.01 |
| Species Oculina | 81.719 | 2.017 | 40.52 | < 0.01 |
| Condition Fe | -29.800 | 1.147 | -25.97 | < 0.01 |
| Contraction Time x Species Oculina | -0.793 | 0.064 | -12.37 | < 0.01 |

**Table S5.** Multivariate analysis of variance (MANOVA) of variable fluorescence parameters and oxygen profiles. Df = degree of freedom; Pillai = Pillai’s trace statistic; Approx F = approximate F-statistic; Num Df = numerator degrees of freedom; Den Df = denominator degrees of freedom.

| *Variable chlorophyll fluorescence* | **Df** | **Pillai** | **Approx F** | **Num DF** | **Den DF** | **p-value** |
| --- | --- | --- | --- | --- | --- | --- |
| Condition | 3 | 0.67696 | 27.17 | 12 | 1119 | < 0.01 |
| Species | 1 | 0.82660 | 442.14 | 4 | 371 | < 0.01 |
| Condition:species | 3 | 0.59904 | 23.27 | 12 | 1119 | < 0.01 |
| Residuals | 374 |  |  |  |  |  |
| *Oxygen profiles* |  |  |  |  |  |  |
| Condition | 3 | 0.41480 | 189.73 | 6 | 4360 | < 0.01 |
| Species | 1 | 0.42135 | 793.32 | 2 | 2179 | < 0.01 |
| Condition:species | 3 | 0.37403 | 167.16 | 6 | 4360 | < 0.01 |
| Residuals | 2180 |  |  |  |  |  |
